# Technology-assisted title and abstract screening for systematic reviews: a retrospective evaluation of the Abstrackr machine learning tool

**DOI:** 10.1186/s13643-018-0707-8

**Published:** 2018-03-12

**Authors:** Allison Gates, Cydney Johnson, Lisa Hartling

**Affiliations:** Alberta Research Centre for Health Evidence, Department of Pediatrics, University of Alberta, 11405-87 Avenue NW, Edmonton, Alberta T6G 1C9 Canada

**Keywords:** Machine learning, Automation, Systematic review, Methodology

## Abstract

**Background:**

Machine learning tools can expedite systematic review (SR) processes by semi-automating citation screening. Abstrackr semi-automates citation screening by predicting relevant records. We evaluated its performance for four screening projects.

**Methods:**

We used a convenience sample of screening projects completed at the Alberta Research Centre for Health Evidence, Edmonton, Canada: three SRs and one descriptive analysis for which we had used SR screening methods. The projects were heterogeneous with respect to search yield (median 9328; range 5243 to 47,385 records; interquartile range (IQR) 15,688 records), topic (Antipsychotics, Bronchiolitis, Diabetes, Child Health SRs), and screening complexity. We uploaded the records to Abstrackr and screened until it made predictions about the relevance of the remaining records. Across three trials for each project, we compared the predictions to human reviewer decisions and calculated the sensitivity, specificity, precision, false negative rate, proportion missed, and workload savings.

**Results:**

Abstrackr’s sensitivity was > 0.75 for all projects and the mean specificity ranged from 0.69 to 0.90 with the exception of Child Health SRs, for which it was 0.19. The precision (proportion of records correctly predicted as relevant) varied by screening task (median 26.6%; range 14.8 to 64.7%; IQR 29.7%). The median false negative rate (proportion of records incorrectly predicted as irrelevant) was 12.6% (range 3.5 to 21.2%; IQR 12.3%). The workload savings were often large (median 67.2%, range 9.5 to 88.4%; IQR 23.9%). The proportion missed (proportion of records predicted as irrelevant that were included in the final report, out of the total number predicted as irrelevant) was 0.1% for all SRs and 6.4% for the descriptive analysis. This equated to 4.2% (range 0 to 12.2%; IQR 7.8%) of the records in the final reports.

**Conclusions:**

Abstrackr’s reliability and the workload savings varied by screening task. Workload savings came at the expense of potentially missing relevant records. How this might affect the results and conclusions of SRs needs to be evaluated. Studies evaluating Abstrackr as the second reviewer in a pair would be of interest to determine if concerns for reliability would diminish. Further evaluations of Abstrackr’s performance and usability will inform its refinement and practical utility.

**Electronic supplementary material:**

The online version of this article (10.1186/s13643-018-0707-8) contains supplementary material, which is available to authorized users.

## Background

Systematic reviews (SRs) provide the highest level of evidence to inform clinical and policy decisions [1]. Specialized guidance documents [2, 3] aim to ensure that reviewers produce evidence syntheses that are methodologically rigorous and transparently reported. The completion of SRs while adhering to strict conduct and reporting standards requires highly skilled individuals, a large time commitment, and substantial financial and material resources. As the standards for the conduct and reporting of SRs have become more stringent [4], they have become more labor-intensive to produce. On average, after registering a protocol, it takes author teams more than 1 year of work before their SR is published [5]. The disconnect between the work required to complete a SR and the rate of publication of new evidence from trials [6] means that many SRs are out of date before they are published [7].

Although the tasks associated with undertaking a SR have been streamlined over recent decades, there remains a clear need for updated methods to produce and update SRs with greater efficiency [8]. These methods will also be applicable to the emerging area of living SRs that seek to keep SRs continuously updated [9, 10]. New technologies hold promise in achieving this mandate while maintaining the rigor associated with traditional SRs. To date, more than 100 software tools have been developed to expedite some of the most time-consuming processes involved in synthesizing evidence [11]. Notably, text mining tools have gained attention for their potential to semi-automate citation screening and selection [12]. Traditionally, human reviewers must screen each record, first by title and abstract, taking at least 30 s per record [13]. The full texts of records accepted at the title and abstract stage then need to be reviewed to come to a decision about their relevance. As search strategies are often highly sensitive but not specific [14], the task can be arduous. Text mining tools can accelerate screening by prioritizing the records most likely to be relevant and eliminating those most likely to be irrelevant [14].

Tools that semi-automate screening are still quite novel and require further development and testing before they can be recommended to complement the work of human reviewers [12, 14]. Presently, nearly 30 software tools developed with the aim of reducing the time to screen records for inclusion in SRs are available [11]. For few, however, does there exist published documentation of their development or evidence of their performance. Many are also not freely accessible, a limitation to their uptake. Before the performance of the various available tools can be compared, there is a need to develop the evidence base for rigorously developed tools that have shown potential. For this reason, we chose to evaluate Abstrackr, a freely available, collaborative, web-based tool that semi-automates title and abstract screening [15]. As of 2012, Abstrackr had been used to facilitate screening in at least 50 SRs [15]. Prospective and retrospective evaluations have provided promising empirical evidence of its performance [15, 16]. With respect to its prediction algorithm, the few existing evaluations have reported screening workload reductions of at least 40% and the incorrect exclusion of few, if any relevant records [15, 16]. Conversely, for some reviews the workload savings have been minimal (< 10%) [16]. Because it is critical that SRs include all relevant data, there also exists legitimate concern that text mining tools may incorrectly exclude relevant records [12].

Abstrackr needs to be tested on screening tasks that vary by size, topic, and complexity [12] to determine its reliability and applicability for a broad range of projects. We therefore undertook a retrospective evaluation of Abstrackr’s ability to semi-automate citation screening for a heterogeneous sample of four screening projects that were completed or ongoing at our center. We measured its performance using standard metrics, including: sensitivity; specificity; precision; false negative rate; proportion missed; and workload savings.

## Methods

### Abstrackr

Abstrackr (http://abstrackr.cebm.brown.edu/) is a freely available online machine learning tool that aims to enhance the efficiency of evidence synthesis by semi-automating title and abstract screening [15]. To begin, the user must upload the records retrieved from an electronic search to Abstrackr’s user interface. The first record is then presented on screen (including the title, abstract, journal, authors, and keywords) and the reviewer is given the option of labeling it as ‘relevant,’ ‘borderline,’ or ‘irrelevant’ using buttons displayed below it. Words (or “terms”) that are indicative of relevance or irrelevance that appear in the titles and abstracts can also be tagged [15]. After the reviewer judges the relevance of the record, the next record appears and the process continues. Abstrackr maintains digital documentation of the labels assigned to each record, which can be accessed at any time. Decisions for the records can be revised if desired. After an adequate sample of records has been screened, Abstrackr presents a prediction regarding the relevance of those that remain.

Details of Abstrackr’s development and of the underlying machine learning technology have been described by Wallace et al. [15]. Briefly, Abstrackr uses text mining to recognize patterns in relevant and irrelevant records, as labeled by the user [16]. Rather than presenting the records in random order, Abstrackr presents records in order of relevance based on a predictive model. Any of the data provided by the user (e.g., labels for the records that are screened and inputted terms) can be exploited by Abstrackr to enhance the model’s performance [15].

### Included screening projects

We selected a convenience sample of four completed or ongoing projects for which title and abstract screening was undertaken at the Alberta Research Centre for Health Evidence (ARCHE), University of Alberta, Canada. The projects were as follows: 1. “Antipsychotics,” a comparative effectiveness review of first and second generation antipsychotics for children and young adults (prepared for the Evidence-based Practice Center (EPC) Program funded by the Agency for Healthcare Research and Quality [AHRQ]) [17]; 2. “Bronchiolitis,” a SR and network meta-analysis of pharmacologic interventions for infants with bronchiolitis (ongoing, PROSPERO: CRD42016048625); 3. “Child Health SRs,” a descriptive analysis of all child-relevant non-Cochrane SRs, meta-analyses, network meta-analyses, and individual patient data meta-analyses published in 2014 (ongoing); and 4. “Diabetes,” a SR of the effectiveness of multicomponent behavioral programs for people with diabetes (prepared for the AHRQ EPC Program) [18, 19]. The sample of projects included a variety of populations, intervention modalities, eligible comparators, outcome measures, and included study types. A description of the PICOS (population, intervention, comparator, outcomes, and study design) characteristics of each project are in Table 1. The screening workload and number of included studies differed substantially between projects (Table 2).

**Table 1:** PICOS (participants, interventions, comparators, outcomes, study design) characteristics of the screening projects

Characteristic	Screening project			
	Bronchiolitis	Antipsychotics	Diabetes	Child Health SRs
Participants	Infants ≤ 24 months	Children and young adults ≤24 years	Any age	Children ≤ 18 years
Intervention	Pharmacologic	Pharmacologic	Multicomponent behavioral program	Any
Comparator	Placebo; active pharmacologic comparator	Placebo; no treatment; active pharmacologic comparator	Usual or standard care; active comparator	Any (including non-comparative SRs)
Outcomes	Rate of admission or length of stay; change in clinical severity score; oxygen saturation; respiratory rate; heart rate; symptoms; QoL; pulmonary function	Intermediate effectiveness outcomes; adverse effects	Behavioral; clinical; health (e.g., quality of life); diabetes-related health care utilization; program acceptability; harms	Health outcomes relevant to children, including the accuracy of diagnostic tests and outcomes measured in adults related to exposures during childhood
Study design	RCTs	RCTs; NRCTs; controlled cohort studies; controlled before-after studies	RCTs; NRCTs; prospective comparative studies; prospective cohort studies; controlled before-after studies	Non-Cochrane: SRs; meta-analyses; network meta-analyses; individual patient data meta-analyses

*NRCT* non-randomized controlled trial, *QoL* quality of life, *RCT* randomized controlled trial, *SR* systematic review

**Table 2:** Screening workload and proportion of records included by screening project, as performed by the human reviewers

Screening characteristics	Screening project (*N* (%))			
	Antipsychotics	Bronchiolitis	Child Health SRs	Diabetes
Records retrieved by the searches	12,763	5893	5243	47,141
Accepted after title and abstract screening^a^	808 (6.3)	520 (8.8)	3143 (59.9)	698 (1.5)
Accepted after full-text screening^b^	135 (1.1)	155 (2.6)	1598 (30.5)	205 (0.4)

*SR* systematic review

^a^Based on dual independent screening by two human reviewers

^b^Records included in the final report

For the SRs, two independent reviewers screened the records retrieved via the electronic searches by title and abstract and marked each as “include,” “unsure,” or “exclude” following a priori screening criteria. The records marked as “include” or “unsure” by either reviewer were eligible for full-text screening. For the descriptive analysis (Child Health SRs), we used an abridged screening method whereby one reviewer screened all titles and abstracts, and a second reviewer only screened the records marked as “unsure” or “exclude.” Akin to the other projects, any records marked as “include” by either reviewer were eligible for full-text screening. The two screening methods were therefore essentially equivalent (although for Child Health SRs we expedited the task by not applying dual independent screening to the records marked as “include” by the first reviewer, as these would automatically move forward to full-text screening regardless of the second reviewer’s decision). In all cases, the reviewers convened to reach consensus on the studies to be included in the final report, making use of a third-party arbitrator when they could not reach a decision.

### Data collection

Our testing began in December 2016 and was completed by September 2017. For each project, the records retrieved from the online searches were stored in one or more EndNote (v. X7, Clarivate Analytics, Philadelphia, PA) databases. We exported these in the form of RIS files and uploaded them to Abstrackr for testing. From Abstrackr’s screening options, we selected “single-screen mode” so that the records would need only to be screened by one reviewer. We also ordered the records as “most likely to be relevant,” so that the most relevant ones would be presented in priority order. We chose the “most likely to be relevant” setting instead of the “random” setting to simulate the method by which Abstrackr may most safely be used [12] by real-world SR teams, whereby it expedites the screening process by prioritizing relevant records. Consistent with previous evaluations [15, 16], we did not tag any terms for relevance or irrelevance.

As the records appeared on screen, one author (AG or CJ) marked each as “relevant” or “irrelevant” based on inclusion criteria for each project. The authors continued screening while checking for the availability of predictions after each 10 records. Once a prediction was available, the authors discontinued screening. We downloaded the predictions and transferred them to a Microsoft Office Excel (v. 2016, Microsoft Corporation, Redmond, WA) workbook. We performed three independent trials per topic to account for the fact that the first record presented to the reviewers appeared to be selected at random. Therefore, the predictions for the same dataset could differ.

### Data analyses

We performed all statistical analyses in IBM SPSS Statistics (v. 24, International Business Machines Corporation, Armonk, NY) and Review Manager (v. 5.3, The Nordic Cochrane Centre, The Cochrane Collaboration, Copenhagen, DK). We described the screening process in Abstrackr using means and standard deviations (SDs) across three trials. To evaluate Abstrackr’s performance, we compared its predictions to the consensus decisions (“include” or “exclude”) of the human reviewers following title and abstract, and full-text screening. We calculated Abstrackr’s sensitivity (95% confidence interval (CI)) and specificity (95% CI) for each trial for each project, and the mean for each project. To ensure comparability to previous evaluations [15, 16], we also calculated descriptive performance metrics using the same definitions and formulae, including precision, false negative rate, proportion missed, and workload savings. We calculated sensitivity, specificity, and the performance metrics using the data from 2 × 2 cross-tabulations for each trial. We defined the metrics as follows, based on previous reports:

- Sensitivity (true positive rate): the proportion of records correctly identified as relevant by Abstrackr out of the total deemed relevant by the human reviewers [20].
- Specificity (true negative rate): the proportion of records correctly identified as irrelevant by Abstrackr out of the total deemed irrelevant by the human reviewers [20].
- Precision: the proportion of records predicted as relevant by Abstrackr that were also deemed relevant by the human reviewers [16].
- False negative rate: the proportion of records that were deemed relevant by the human reviewers that were predicted as irrelevant by Abstrackr [16].
- Proportion missed: the number of records predicted as irrelevant by Abstrackr that were included in the final report, out of the total number of records predicted as irrelevant [16].
- Workload savings: the proportion of records predicted as irrelevant by Abstrackr out of the total number of records to be screened [16] (i.e., the proportion of records that would not need to be screened manually) [15].

Because the standard error (SE) approximated zero in most cases (given the large number of records per dataset), we presented only the calculated value and not the SE for each metric. For each project, we calculated the mean value for each metric across the three trials. We also calculated the SD for the mean of the range of values observed across the trials.

We counted the total number of records included within the final report that were predicted as irrelevant by Abstrackr. We estimated the potential time saved (hours and days), assuming a screening rate of 30 s per record [13] and an 8-h work day. Additional file 1 shows an example of the 2 × 2 cross-tabulations and sample calculations for each metric.

## Results

### Descriptive characteristics of the screening process

Table 3 shows the characteristics of the title and abstract screening processes in Abstrackr. Details of each trial are in Additional file 2. Comparing the four projects, we needed to screen the fewest records for Child Health SRs (mean (SD), 210 (10)) and the most for Bronchiolitis (607 (340)) before Abstrackr made predictions. The mean (SD) human screening workload for Antipsychotics and Diabetes was 277 (32) and 323 (206) records, respectively. By the proportion of total records to be screened, we had to screen the fewest records for Diabetes (0.7 (0.4)% of the 47,385 records) and the most for Bronchiolitis (10.3 (5.8)% of the 5893 records) before Abstrackr made predictions. The mean (SD) proportion of records we had to screen for Antipsychotics and Child Health SRs was 2.2 (0.3)% (of 12,763 records) and 4.0 (0.2)% (of 5243 records), respectively.

**Table 3:** Descriptive characteristics of the title and abstract screening processes in Abstrackr, across three trials

Characteristic	Topic			
	Antipsychotics *N* = 12,763 records	Bronchiolitis *N* = 5893 records	Child Health SRs *N* = 5243 records	Diabetes *N* = 47,385 records^a^
Screened by human^b^				
N records	277 (32)	607 (340)	210 (10)	323 (206)
% records	2.2 (0.3)	10.3 (5.8)	4.0 (0.2)	0.7 (0.4)
Accepted by human^c^				
N records	19 (3)	56 (35)	118 (20)	111 (74)
% records	6.9 (1.1)	9.0 (0.9)	56.1 (6.9)	34.1 (1.6)
Predicted as relevant by Abstrackr^d^				
N records	4259 (1281)	1163 (123)	4535 (173)	5187 (1430)
% records	34.1 (10.2)	22.0 (0.9)	90.1 (3.6)	11.0 (3.0)

All values are mean (SD) across three trials. Standard deviations for proportions (% records) relate to the range of values observed across trials, and not the mean variance across trials

*SR* systematic review

^a^Included some duplicates as three EndNote libraries were combined to create the dataset

^b^Before Abstrackr produced predictions

^c^Based on the decisions of two independent human reviewers for each screening project

^d^Records that Abstrackr predicted as relevant for further inspection following title and abstract screening (equivalent to “accepted as relevant”)

Of the remaining records to be screened, Abstrackr predicted on average (SD) that 90.1 (3.6)% (4536 (173) records) of those for Child Health SRs were relevant for further inspection, compared to just 11.0 (3.0)% (5187 (1430) records) of those for Diabetes. The proportion predicted relevant for Antipsychotics and Bronchiolitis were 34.1 (10.2)% (4259 (1281) records) and 22.0 (0.9)% (1163 (123) records), respectively.

### Sensitivity and specificity

**Fig. 1:**
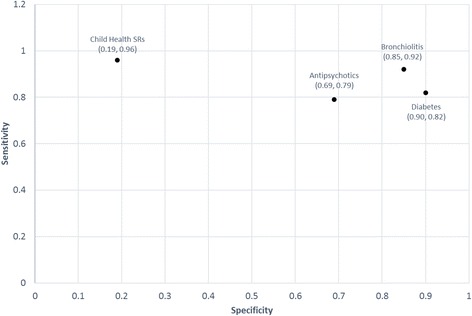
Abstrackr’s mean sensitivity and specificity across three trials for each project

Figure 1 shows Abstrackr’s sensitivity and specificity for the four projects. On average, Abstrackr’s sensitivity was best for Child Health SRs (0.96) followed by Bronchiolitis (0.92), Diabetes (0.82), and Antipsychotics (0.79). Abstrackr’s specificity was best for Diabetes (0.90) followed by Bronchiolitis (0.85), Antipsychotics (0.69), and Child Health SRs (0.19). Details of the sensitivity and specificity for the individual trials for each project are in Additional file 3.

### Performance metrics

Table 4 shows a comparison of Abstrackr’s performance (based on the standard metrics) for the four projects. Abstrackr’s mean (SD) precision was best for Child Health SRs (64.7 (2.0)%) followed by Bronchiolitis (38.1 (2.6)%), Antipsychotics (15.1 (2.6)%), and Diabetes (14.8 (2.6)%). The false negative rate was highest for Antipsychotics (21.2 (8.3)%) followed by Diabetes (17.9 (2.3)%), Bronchiolitis (7.3 (2.2)%) and Child Health SRs (3.5 (1.4)%). The proportion missed was highest for Child Health SRs (6.4 (1.7)%). For Antipsychotics and Bronchiolitis, the proportion missed was 0.1 (0.1)%. For Diabetes, the proportion missed was 0.1 (0.01)%. The workload savings was largest for Diabetes (88.4 (2.7)%) followed by Antipsychotics (64.5 (9.8)%), Bronchiolitis (70.0 (3.7)%) and Child Health SRs (9.5 (3.5)%). Details of the precision, false negative rate, proportion missed, and workload savings for the individual trials for each project are in Additional file 4.

**Table 4:** Abstrackr’s mean performance across three trials for each of the screening projects

Performance metric	Topic			
	Antipsychotics *N* = 12,763 records	Bronchiolitis *N* = 5893 records	Child Health SRs *N* = 5243 records	Diabetes *N* = 47,385 records^a^
Precision, % (SD)	15.1 (2.6)	38.1 (2.6)	64.7 (2.0)	14.8 (2.6)
False negative rate, % (SD)	21.2 (8.3)	7.3 (2.2)	3.5 (1.4)	17.9 (2.3)
Proportion missed, % (SD)	0.1 (0.1)	0.1 (0.1)	6.4 (1.7)	0.1 (0.01)
Workload savings, % (SD)	64.5 (9.8)	70.0 (3.7)	9.5 (3.5)	88.4 (2.7)

Standard deviations for proportions relate to the range of values observed across trials and not the mean variance across trials

*SD* standard deviation

^a^Included some duplicates, as three EndNote libraries were combined to create the dataset

### Records missed and time savings

Across the three trials, Abstrackr missed 16, 20, and 25 (7.8, 9.8, and 12.2%) of the studies included in the final SR for Diabetes; 1, 13, and 14 (0.7, 9.6, and 10.4%) of the studies for Antipsychotics; 0, 5, and 8 (0, 3.2, and 5.2%) of the studies for Bronchiolitis; and 24, 31, and 35 (1.5, 1.9, and 2.2%) of the studies for Child Health SRs. Based on an estimate of 30 s of screening time per record and 8 work hours per day, the largest time savings was for Diabetes (349 h or 44 days) followed by Antipsychotics (69 h or 9 days), Bronchiolitis (34 h or 4 days), and Child Health SRs (4 h or 0.5 days).

## Discussion

Abstrackr’s ability to predict relevant records was previously evaluated by Wallace et al. [15] in 2012 and Rathbone et al. [16] in 2015. Both author groups reported impressive reductions in reviewer workload (~ 40%), and few incorrect predictions for studies included in the final reports [15, 16]. We have added to these findings by investigating Abstrackr’s reliability for a heterogeneous sample of screening projects. The program’s sensitivity exceeded 0.75 for all screening tasks, while specificity and precision, the proportion missed, and the workload savings varied. Our findings for the descriptive analysis are novel and suggest that Abstrackr cannot always reliably distinguish between relevant and irrelevant records. Moreover, this example shows that for certain screening tasks, Abstrackr may not infer practically significant reductions in screening workload. As opposed to previous evaluations [15, 16], we undertook three trials of Abstrackr for each screening task. Performance varied, albeit relatively minimally, between each trial.

Abstrackr’s precision ranged from 15 to 65% and was lower for Antipsychotics and Diabetes compared to Bronchiolitis and Child Health SRs. Our findings support those of Rathbone et al. [16], who found that precision was affected by the complexity of the inclusion criteria and the ratio of included records to the screening workload. With respect to the inclusion criteria, for Antipsychotics, the population of interest included children and young adults. Because “young adults” and “adults” are not mutually exclusive categories, relevant and irrelevant records are difficult for a text mining tool to distinguish, likely contributing to lower precision. The screening criteria for Antipsychotics and Diabetes were also more complex because the SRs aimed to answer multiple key questions. For Child Health SRs, only SRs were included, but the term “systematic review” is often inaccurately used, a nuance more easily picked up by humans than by a machine. With respect to the proportion of included records, for Diabetes, the screening workload was large while the proportion of included studies was small. Comparatively, the screening workload for Child Health SRs was small, but the proportion of included records was large. It is likely that screening tasks that contain a large proportion of irrelevant records are more difficult to semi-automate. Moreover, supervised machine learning is known to perform better on larger datasets [16].

Abstrackr’s false negative rate varied, ranging from 3.5 to 21.2%. The proportion missed was just 0.1% for all SRs. Consistent with reports of this and other text mining tools for citation selection [12], this equated to a median of 4.2% (range 0 to 12.2%; IQR 7.8%) of the records included in the final reports. Of note, citation screening by human reviewers is not perfect. Edwards et al. (2012) found that single reviewers missed on average 9% of relevant records per screening task [21]. The proportion of records missed for two reviewers who reached consensus, however, was a negligible 0 to 1% [21]. Accordingly, Cochrane standards [3] require two human reviewers to screen records independently for eligibility. Within this context, as a means to eliminate irrelevant records Abstrackr’s performance is akin to that of a single human reviewer, but suboptimal in most cases compared to the consensus two reviewers.

For the SRs, Abstrackr reduced the number of records to screen by 65 to 88%, which would represent sizeable time savings, especially for reviews where large numbers of records were retrieved via the searches. This gain in efficiency came at the cost of potentially omitting relevant records. Even when the proportion of missed records is small, excluding key studies could seriously bias effect estimates [22], resulting in misleading conclusions. For Child Health SRs, the workload savings was just 9.5%. The screening task for this project was atypical; the search was limited to SRs but the inclusion criteria were broad and unrestricted by condition, intervention, comparator, or outcome. Accordingly, 59.9% of the records were accepted following title and abstract screening, compared to the median 2.9% in health-related SRs [23]. It is possible that Abstrackr may be better suited to screening projects with narrower research questions.

### Implications for research and practice

Owing to the potential for missing relevant records, and to variations in performance by screening task, further development and testing of Abstrackr on a broad range of projects is required before it can be recommended to reduce screening workloads. Bekhuis et al. [24–26] found that employing machine learning tools as the second screener in a reviewer pair could overcome concerns about reliability. Prospective studies evaluating Abstrackr’s performance as the second reviewer in a pair are required to confirm or refute if the tool is suitable for such a task. The knowledge and screening experience of the human reviewer would be important to consider, with preference given to highly competent content experts to reduce the likelihood that records predicted as irrelevant would be overlooked. Future evaluations should investigate whether the missed records would affect the results or conclusions of the SR, or if these would be located via other means, e.g., cited reference search, contacting authors.

Along with accumulating evidence of Abstrackr’s performance, there is a need for usability data [12] to determine the acceptability and practicality of the tool in real-world evidence synthesis projects. Although we did not set out to investigate these qualities, anecdotally we encountered some difficulty successfully uploading the records and obtaining the predictions in Abstrackr. The time lost troubleshooting these issues detracted from the workload savings achieved once the technical issues were overcome. Information about user experiences could be used to enhance the practical appeal of machine learning tools, which will be necessary if they are to be incorporated into everyday practices.

### Strengths and limitations

Our study adds to the limited data on Abstrackr’s performance and to the growing body of research on text mining and machine learning tools for citation selection [12]. Within our heterogeneous sample of screening tasks Abstrackr’s performance varied, so the findings should not be generalized. We also noted the potential for variation in predictions between trials of the same screening task. We could not control the first record to be screened, which influenced the prioritization of subsequent records and the resulting predictions.

Of note, we used the “most likely to be relevant” setting in Abstrackr to prioritize the most relevant records for screening. It is possible that if we had used the “random” setting that our findings would have differed. We relied on the gold-standard “include” and “exclude” decisions of human reviewers to train the tool. In real-life evidence synthesis projects, two reviewers screen the records independently, some records are classified as “unsure,” and the reviewers do not always agree. We are uncertain to what extent evaluating the tool prospectively would have impacted Abstrackr’s predictions.

## Conclusions

For a heterogeneous sample of four screening projects, Abstrackr reliability was variable. The workload savings were minimal for some projects and substantial for others, and appeared to depend on the qualities of the screening task. Reducing the screening workload came at the expense of potentially omitting relevant records. The extent to which missing records might affect the results or conclusions of the SRs, or whether using Abstrackr as a second reviewer could reduce reliability concerns remain to be investigated. Nevertheless, Abstrackr performed as well in most cases as a single human reviewer. Further research is required to evaluate Abstrackr’s performance on a diversity of screening tasks and to determine its usability. Such evaluations will serve to refine the tool and inform its practical utility for real-world evidence synthesis tasks.

## Additional files

Additional file 1: Sample calculations for sensitivity, specificity, and performance metrics. Sample calculations for sensitivity, specificity, and performance metrics. (DOCX 17 kb)

Additional file 2: Descriptive characteristics of the title and abstract screening processes in Abstrackr. Additional table showing the descriptive characteristics of the title and abstract screening processes in Abstrackr. (DOCX 16 kb)

Additional file 3: Abstrackr’s sensitivity and specificity across three trials and overall. Additional table showing Abstrackr’s sensitivity and specificity across three trials for each project and overall. (DOCX 15 kb)

Additional file 4: Performance metrics for each trial and overall by screening project. Additional table showing the performance metrics for each trial and overall by screening project. (DOCX 16 kb)

## Abbreviations

AHRQ: Agency for Healthcare Research and Quality
ARCHE: Alberta Research Centre for Health Evidence
CI: Confidence Interval
EPC: Evidence-based Practice Center
IQR: Interquartile range
PROSPERO: International prospective register of systematic reviews
SD: Standard deviation
SE: Standard error
SR: Systematic review
