# Supplementary material for: Technology-assisted title and abstract screening for systematic reviews: a retrospective evaluation of the Abstrackr machine learning tool
Source: Syst Rev. 2018 Mar 12;7:45. doi: 10.1186/s13643-018-0707-8 (PMC5848519; doi:10.1186/s13643-018-0707-8)
Supplement: Supplementary file 1 — Sample calculations for sensitivity, specificity, and performance metrics. Sample calculations for sensitivity, specificity, and performance metrics. (DOCX 17 kb) [file 13643_2018_707_MOESM1_ESM.docx]

**2x2 Cross-tabulations for Child Health SRs, Trial 1**

*Title and abstract screening*

|  |  | **Human consensus decision** | | **Total** |
| --- | --- | --- | --- | --- |
|  |  | **Exclude** | **Include** |  |
| **Abstrackr prediction** | **Exclude** | 540 | 150 | 690 |
|  | **Include** | 1442 | 2911 | 4353 |
| **Total** | | 1982 | 3061 | 5043 |

*Full text screening (i.e., records included in the final report)*

|  |  | **Human consensus decision** | | **Total** |
| --- | --- | --- | --- | --- |
|  |  | **Exclude** | **Include** |  |
| **Abstrackr prediction** | **Exclude** | 659 | 31 | 690 |
|  | **Include** | 2816 | 1537 | 4353 |
| **Total** | | 3475 | 1568 | 5043 |

Note: Predictions were available after screening 200 records. The total records to be screened was 5243.

**Sample Calculations**

**SENSITIVITY:** *The proportion of records correctly identified as relevant by Abstrackr out of the total deemed relevant by the human reviewers (following title and abstract screening)*

Sensitivity = (true positives) / (true positives + false negatives) = 2911 / 2911 + 150 = 0.95

**SPECIFICITY:** *The proportion of records correctly identified as irrelevant by Abstrackr out of the total deemed irrelevant by the human reviewers (following title and abstract screening)*

Specificity = (true negatives) / (true negatives + false positives) = 540 / 540 + 1442 = 0.27

**PRECISION:** *The proportion of records predicted as relevant by Abstrackr that were also deemed relevant by the human reviewers (following title and abstract screening)*

Precision = # records correctly identified as relevant / all records identified as relevant

= 2911 / 4353 = 66.9%

**FALSE NEGATIVE RATE:** *The proportion of records that were deemed relevant by the human reviewers that were predicted as irrelevant by Abstrackr (following title and abstract screening)*

False negative rate = # records incorrectly identified as irrelevant / all records identified as relevant

= 150 / 3061 = 4.9%

**PROPORTION MISSED:** *The number of records predicted as irrelevant by Abstrackr that were included in the final report, out of the total number of records predicted as irrelevant*

Proportion missed = # incorrectly predicted as irrelevant / all records predicted as irrelevant

= 31 / 690 = 4.5%

**WORKLOAD SAVINGS:** *The proportion of records predicted as irrelevant by Abstrackr out of the total number of citations to be screened (i.e., the proportion of citations that would not need to be screened manually)*

Workload savings = # records predicted as irrelevant / total # records to be screened

= 690 / 5243 = 13.2%

**TIME SAVINGS:** *Time saved based on the records that would not need to be screened (i.e., those predicted as irrelevant by Abstrackr); estimated based on a screening rate of 0.5 minutes per record and an 8-hour work day*

Time savings = [(# records predicted as irrelevant x 0.5 min/record) / 60 (min/ h)] / (8 h/day)

= (690 x 0.5) / 60 min/h = 5.75 h / (8h/day) = 0.7 days
