# Supplementary material for: Technology-assisted title and abstract screening for systematic reviews: a retrospective evaluation of the Abstrackr machine learning tool
Source: Syst Rev. 2018 Mar 12;7:45. doi: 10.1186/s13643-018-0707-8 (PMC5848519; doi:10.1186/s13643-018-0707-8)
Supplement: Supplementary file 2 — Descriptive characteristics of the title and abstract screening processes in Abstrackr. Additional table showing the descriptive characteristics of the title and abstract screening processes in Abstrackr. (DOCX 16 kb) [file 13643_2018_707_MOESM2_ESM.docx]

**Additional Table 1.** Descriptive characteristics of the title and abstract screening processes in Abstrackr

| **Characteristic** | **Trial 1 (N (%))** | **Trial 2 (N (%))** | **Trial 3 (N (%))** | **Mean (N (SD))** | **Mean % (SD)**^a^ |
| --- | --- | --- | --- | --- | --- |
| **Antipsychotics (12763 records)** | | | | | |
| Screened by human | 300 (2.4) | 240 (1.9) | 290 (2.3) | 277 (32) | 2.2 (0.3) |
| Accepted by human | 17 (5.7) | 17 (7.1) | 23 (7.9) | 19 (3) | 6.9 (1.1) |
| Predicted relevant by Abstrackr^b^ | 3532 (28.3) | 5738 (45.8) | 3508 (28.1) | 4259 (1281) | 34.1 (10.2) |
| **Bronchiolitis (5893 records)** | | | | | |
| Screened by human | 890 (15.1) | 700 (11.9) | 230 (3.9) | 607 (340) | 10.3 (5.8) |
| Accepted by human | 89 (10.0) | 58 (8.3) | 20 (8.7) | 56 (35) | 9.0 (0.9) |
| Predicted relevant by Abstrackr | 1080 (21.6) | 1104 (21.3) | 1304 (23.0) | 1163 (123) | 22.0 (0.9) |
| **Child Health SRs (5243 records)** | | | | | |
| Screened by human | 200 (3.8) | 220 (4.2) | 210 (4.0) | 210 (10) | 4.0 (0.2) |
| Accepted by human | 98 (49.0) | 138 (62.7) | 119 (56.7) | 118 (20) | 56.1 (6.9) |
| Predicted relevant by Abstrackr | 4353 (86.3) | 4697 (93.5) | 4557 (90.5) | 4536 (173) | 90.1 (3.6) |
| **Diabetes (47385 records)**^c^ | | | | | |
| Screened by human | 540 (1.1) | 300 (0.6) | 130 (0.3) | 323 (206) | 0.7 (0.4) |
| Accepted by human | 191 (35.4) | 97 (32.3) | 45 (34.6) | 111 (74) | 34.1 (1.6) |
| Predicted relevant by Abstrackr | 4497 (9.6) | 4232 (9.0) | 6831 (14.5) | 5187 (1430) | 11.0 (3.0) |

SD = standard deviation; SR = systematic review

^a^Standard deviations for proportions (% records) relate to the range of values observed across trials, and not the mean variance across trials.

^b^Records that Abstrackr predicted as relevant for further inspection following title and abstract screening (equivalent to “accepted as relevant”).

^c^Included some duplicates, as three EndNote libraries were combined to create the dataset.
