# Supplementary material for: Technology-assisted title and abstract screening for systematic reviews: a retrospective evaluation of the Abstrackr machine learning tool
Source: Syst Rev. 2018 Mar 12;7:45. doi: 10.1186/s13643-018-0707-8 (PMC5848519; doi:10.1186/s13643-018-0707-8)
Supplement: Supplementary file 3 — Abstrackr’s sensitivity and specificity across three trials and overall. Additional table showing Abstrackr’s sensitivity and specificity across three trials for each project and overall. (DOCX 15 kb) [file 13643_2018_707_MOESM3_ESM.docx]

**Additional table 2.** Abstrackr’s sensitivity and specificity across three trials and overall

| **Performance metric** | **Trial 1 (95% CI)** | **Trial 2 (95% CI)** | **Trial 3 (95% CI)** | **Mean of 3 trials** |
| --- | --- | --- | --- | --- |
| **Antipsychotics (12763 records)** | | | | |
| Sensitivity^a^ | 0.73 (0.70, 0.76) | 0.88 (0.86, 0.90) | 0.75 (0.72, 0.78) | 0.79 |
| Specificity^b^ | 0.75 (0.74, 0.75) | 0.57 (0.56, 0.58) | 0.75 (0.74, 0.76) | 0.69 |
| **Bronchiolitis (5893 records)** | | | | |
| Sensitivity | 0.91 (0.88, 0.94) | 0.95 (0.93, 0.97) | 0.91 (0.89, 0.94) | 0.92 |
| Specificity | 0.85 (0.84, 0.86) | 0.86 (0.85, 0.87) | 0.84 (0.83, 0.85) | 0.85 |
| **Child Health Systematic Reviews (5243 records)** | | | | |
| Sensitivity | 0.95 (0.94, 0.96) | 0.98 (0.97, 0.98) | 0.96 (0.96, 0.97) | 0.96 |
| Specificity | 0.27 (0.25, 0.29) | 0.13 (0.12, 0.15) | 0.18 (0.17, 0.20) | 0.19 |
| **Diabetes (47385 records)** | | | | |
| Sensitivity | 0.82 (0.80, 0.85) | 0.80 (0.77, 0.82) | 0.84 (0.82, 0.86) | 0.82 |
| Specificity | 0.92 (0.91, 0.92) | 0.92 (0.92, 0.93) | 0.87 (0.87, 0.87) | 0.90 |

CI = confidence interval

^a^The proportion of records correctly identified as relevant by Abstrackr out of the total deemed relevant by the human reviewers.

^b^The proportion of records correctly identified as irrelevant by Abstrackr out of the total deemed irrelevant by the human reviewers.
