# Supplementary material for: Technology-assisted title and abstract screening for systematic reviews: a retrospective evaluation of the Abstrackr machine learning tool
Source: Syst Rev. 2018 Mar 12;7:45. doi: 10.1186/s13643-018-0707-8 (PMC5848519; doi:10.1186/s13643-018-0707-8)
Supplement: Supplementary file 4 — Performance metrics for each trial and overall by screening project. Additional table showing the performance metrics for each trial and overall by screening project. (DOCX 16 kb) [file 13643_2018_707_MOESM4_ESM.docx]

**Additional table 3.** Performance metrics for each trial and overall by screening project

| **Performance metric** | **Trial 1 (%)** | **Trial 2 (%)** | **Trial 3 (%)** | **Mean % (SD)**^a^ |
| --- | --- | --- | --- | --- |
| **Antipsychotics (12763 records)** | | | | |
| Precision | 16.3 | 12.2 | 16.9 | 15.1 (2.6) |
| False negative rate | 27.1 | 11.7 | 24.7 | 21.2 (8.3) |
| Proportion missed | 0.2 | 0.01 | 0.2 | 0.1 (0.1) |
| Workload savings | 70.0 | 53.2 | 70.2 | 64.5 (9.8) |
| **Bronchiolitis (5893 records)** | | | | |
| Precision | 37.7 | 40.9 | 35.7 | 38.1 (2.6) |
| False negative rate | 8.5 | 4.9 | 8.6 | 7.3 (2.2) |
| Proportion missed | 0.1 | 0.0 | 0.2 | 0.1 (0.1) |
| Workload savings | 66.6 | 69.4 | 74.0 | 70.0 (3.7) |
| **Child Health Systematic Reviews (5243 records)** | | | | |
| Precision | 66.9 | 63.0 | 64.3 | 64.7 (2.0) |
| False negative rate | 4.9 | 2.1 | 3.7 | 3.5 (1.4) |
| Proportion missed | 4.5 | 7.4 | 7.4 | 6.4 (1.7) |
| Workload savings | 13.2 | 6.2 | 9.1 | 9.5 (3.5) |
| **Diabetes (47385 records)**^b^ | | | | |
| Precision | 15.1 | 17.2 | 12.0 | 14.8 (2.6) |
| False negative rate | 17.6 | 20.3 | 15.8 | 17.9 (2.3) |
| Proportion missed | 0.04 | 0.05 | 0.06 | 0.1 (0.01) |
| Workload savings | 89.4 | 90.4 | 85.3 | 88.4 (2.7) |

^a^Standard deviations for the proportions relate to the range of values observed across trials, and not the mean variance across trials.

^b^Included some duplicates, as three EndNote libraries were combined to create the dataset.
